# Perioperative Blood Transfusion is Associated with Postoperative Systemic Inflammatory Response and Poorer Outcomes Following Surgery for Colorectal Cancer

**DOI:** 10.1245/s10434-019-07984-7

**Published:** 2019-10-29

**Authors:** Stephen T. McSorley, Alexander Tham, Ross D. Dolan, Colin W. Steele, Jason Ramsingh, Campbell Roxburgh, Paul G. Horgan, Donald C. McMillan

**Affiliations:** Academic Unit of Surgery, University of Glasgow, Glasgow, UK

## Abstract

**Background:**

The present study investigated relationships between perioperative blood transfusion, postoperative systemic inflammatory response, and outcomes following surgery for colorectal cancer.

**Methods:**

Data were recorded for patients (*n* = 544) undergoing potentially curative, elective surgery for colorectal cancer at a single center between 2012 and 2017. Transfusion history was obtained retrospectively from electronic records. Associations between blood transfusion, postoperative C-reactive protein (CRP), albumin, hemoglobin, complications, cancer-specific survival and overall survival (OS) were assessed using propensity score matching (*n *=116).

**Results:**

Of 544 patients, the majority were male (*n *=294, 54%), over 65 years of age (*n *=350, 64%), and with colonic (*n *=347, 64%) node-negative disease (*n *=353, 65%). Eighty-six patients (16%) required perioperative blood transfusion. In the unmatched cohort, blood transfusion was associated with higher median postoperative day (POD) 3 CRP {143 [interquartile range (IQR) 96–221 mg/L] vs. 120 (IQR 72–188 mg/L); *p* = 0.004}, lower median POD 3 albumin [24 (IQR 20–26 g/L) vs. 27 (IQR 24–30 g/L); *p* < 0.001], more postoperative complications [odds ratio (OR) 3.28, 95% confidence interval (CI) 2.03–5.29] and poorer OS [hazard ratio (HR) 3.18, 95% CI 2.08–4.84]. In the propensity score matched cohort, blood transfusion was similarly associated with higher median POD 3 CRP [130 (IQR 93–196 mg/L) vs. 113 (IQR 66–173 mg/L); *p* = 0.046], lower median POD 3 albumin [24 (IQR 20–26 g/L) vs. 26 (IQR 24–30 g/L); *p* < 0.001], more postoperative complications (OR 2.91, 95% CI 1.36–6.20) and poorer OS (HR 2.38, 95% CI 0.99–5.73).

**Conclusions:**

Perioperative blood transfusion was associated with postoperative inflammation, complications, and poorer survival in patients undergoing colorectal cancer surgery, with and without propensity score techniques.

A significant proportion of patients undergoing surgery for colorectal cancer require allogeneic blood transfusion in the perioperative period.^1^ Such transfusions are associated with infective postoperative complications and anastomotic leak.^2^,^3^ They are also associated with disease recurrence,^3^,^4^ and this effect is even greater in the presence of infective complications.^5^ Therefore, it has long been hypothesized that allogeneic blood transfusion might impair the host adaptive immune response to both pathogens and circulating or micrometastatic tumor cells.^6^

There is increasing evidence that an exaggerated postoperative systemic inflammatory response following surgery for colorectal cancer is associated with postoperative complications and long-term survival.^7^ Furthermore, there is some observational evidence that modulation of this response can improve both short-8, and long-term outcomes.^9^ It may be that perioperative blood transfusion and postoperative complications have a negative impact on oncologic outcomes via a common pathway, the systemic inflammatory response.^10^

The aim of this study was to examine the association between perioperative blood transfusion and the magnitude of postoperative systemic inflammatory response, in particular C-reactive protein (CRP) and albumin, which have been reported to be reliable indicators of the magnitude of surgical injury.^11^ Furthermore, the study aimed to examine the impact of blood transfusion on the development of postoperative complications and long-term outcomes following surgery for colorectal cancer.

## Materials and Methods

### Patients

This observational study was a retrospective analysis of a prospectively collected and maintained cohort. Inclusion criteria were patients from a single surgical unit with colorectal cancer who underwent surgery with curative intent between January 2012 and June 2017. Exclusion criteria were emergency surgery, palliative presentation, pre-existing systemic inflammatory comorbidity, hematological malignancy, and long-term steroids.

Prior to and following surgery, all cases were discussed at a multidisciplinary colorectal oncology meeting. Antimicrobial and venous thromboembolism prophylaxis was administered at the time of surgery. Perioperative care was standardized using an ‘enhanced recovery’ protocol. A proportion of patients received postoperative nausea and vomiting prophylaxis in the form of intravenous dexamethasone during surgery, at the discretion of the consultant anesthetist.^8^,^12^

During the study period, there was no formal perioperative blood transfusion protocol. As reported previously,^13^ between June 2016 and June 2017 a preoperative iron replacement protocol was in place for the relatively small number of patients found to have true iron-deficient anemia.

On each day from surgery to discharge, patients had a bedside review by the responsible operating team, and blood samples were drawn for CRP and albumin. The patient’s surgical team arranged further investigation, and/or intervention, based on clinical assessment and these blood results.

## Methods

Patient data were recorded securely and anonymized prior to analysis. The data collected included factors previously reported to be associated with the postoperative systemic inflammatory response, including modified Glasgow Prognostic Score (mGPS), laparoscopic resection, body mass index (BMI) and American Society of Anesthesiologists (ASA) score.^14^ TNM stage (TNM, 5th ed American Joint Committee on Cancer [AJCC]) and pathological features were recorded, as were demographic variables.

Hematological parameters were recorded from full blood count samples taken within 2 weeks prior to surgery. Patients were classified as having anemia based on World Health Organization (WHO) guidelines for males, i.e. hemoglobin (Hb) < 130 mg/L, and females, i.e. Hb < 120 mg/L.^15^ Furthermore, anemic patients were classified as having microcytic anemia with mean corpuscular volume (MCV) < 80 f/L, normocytic anemia with MCV 80–100 f/L, or macrocytic anemia with MCV > 100 f/L.

Retrospective linkage with the blood transfusion electronic database allowed recording of the transfusion of packed red cells (PRCs), number of units transfused, and the time given. Perioperative blood transfusion was defined as any transfusion of PRCs from 30 days prior to 30 days following surgery. All units of PRCs were leucodepleted following screening for blood-borne viruses (HIV, Human T-lymphotropic virus, and hepatitis B and C), malaria, and syphilis. All PRCs were stored between 2 and 6 °C and used within 35 days of donation.

Serum concentrations of CRP (mg/L) were measured using an autoanalyzer (Architect; Abbot Diagnostics, Maidenhead, UK) with a lower detectable limit of 0.2 mg/L, as was serum albumin (normal range 35–50 g/L). The mGPS was calculated in patients for whom preoperative serum CRP and albumin were available.^16^ On days 3 and 4, after surgery for those patients with available CRP and albumin, the validated postoperative Glasgow Prognostic Score (poGPS) was calculated.^7^

If complications occurred between surgery and first clinic follow-up, they were recorded according to the most severe, using the Clavien–Dindo grade,^17^ and type. Infective complications were categorized as described previously.^18^ Superficial surgical site infection was defined as the presence of pus either drained from the wound or discharging spontaneously. Deep surgical site infection was diagnosed either on imaging, radiological drainage, or surgical drainage of intra-abdominal pus, while anastomotic leak was diagnosed at laparotomy or on cross-sectional imaging. Lower respiratory tract infection was defined as consolidation on chest radiograph or computed tomography (CT) scan associated with pyrexia and requiring antibiotic treatment, whereas septicemia was defined as a combination of the systemic inflammatory response syndrome and positive blood culture. Urinary tract- or catheter-related infection was only diagnosed in the presence of the systemic inflammatory response syndrome and positive urine culture.

The study was approved by the West of Scotland Research Ethics Committee, Glasgow.

### Statistical Analysis

In the unmatched cohort, categorical data were compared using the Chi square test and Chi square for linear association where appropriate. Postoperative CRP, albumin, and Hb were non-normal, presented as medians and ranges, and compared using the Mann–Whitney *U* test. The magnitude of CRP, albumin, and Hb by each postoperative day (POD) was displayed graphically as 95% confidence intervals of the median.

Following exclusion of deaths within 30 days of surgery or during the index admission, cancer-specific survival (CSS) was defined as the time from the day of resection to the day of colorectal cancer death. Overall survival (OS) was defined as the time from the day of resection to that of all-cause death. Survival was presented as a cumulative proportion of patients alive at 5 years following surgery and assessed using the log-rank test.

Multivariate logistic regression generated propensity scores, predicting the probability of having received a perioperative transfusion, based on variables associated with the postoperative systemic inflammatory response or complications^14^; age, sex, BMI, ASA, mGPS, tumor site, TNM stage, neojuvant chemoradiotherapy, surgical approach (open or laparoscopic), administration of intraoperative dexamethasone, and preoperative anemia. Patients who received a perioperative blood transfusion were then matched 1:1 with a patient who did not, using the closest propensity score on the logit scale (calliper < 0.05, order of match selection randomized, no replacement). Categorical data were compared using McNemar’s test, while continuous data were compared using the related samples Wilcoxon signed-rank test. The appropriateness of the propensity score matching was assessed visually by the frequency of propensity scores in each group before and after matching.

Sensitivity analyses compared the effect size of perioperative blood transfusion in relation to CRP concentrations on postoperative day 3 using linear regression, postoperative complications using binary logistic regression presented as odd ratios (OR) and 95% CI, and overall survival using Cox regression presented as hazard ratios (HR) and 95% CI, between the unmatched cohort, propensity score matched cohort, and using propensity score regression.

Factors associated with postoperative complications and OS among patients receiving perioperative blood transfusion were examined using multivariate binary logistic regression and multivariate Cox regression, respectively. Variables associated with outcomes at a univariate level of *p* < 0.1 were entered into a backward conditional model, where *p* < 0.05 was considered statistically significant.

In all tests, a two-sided *p* value < 0.05 was considered statistically significant. Propensity scoring matching and all statistical analyses were performed using IBM SPSS version 24 for Windows (IBM Corporation, Armonk, NY, USA).

## Results

### Patients

Overall, 544 patients were included, the majority of whom were male (*n *=294, 54%), over 65 years of age (*n *=350, 64%), and with colonic (*n *=347, 64%) and node-negative disease (*n *=353, 65%). One hundred and ninety patients (35%) were anemic prior to surgery, of whom 42 (8%) had microcytic anemia and 148 (27%) had normocytic anemia. Eighty-six patients (16%) required allogeneic blood transfusion within the perioperative period, with 9 patients (2%) receiving a preoperative blood transfusion, 67 (12%) receiving an intraoperative or postoperative blood transfusion, and 10 (2%) receiving a transfusion at multiple time points. Two hundred and seven patients (38%) experienced a complication, of which 130 (24%) were infective, with 23 (4%) anastomotic leaks, and 53 (10%) were Clavien–Dindo grade 3–5. There were 7 (1%) deaths within 30 days of surgery. During the follow-up period, 87 patients died (16%), 52 (10%) cases of which were due to colorectal cancer. The median follow-up of those alive at the time of censoring was 43 months (range 12–77).

### Perioperative Blood Transfusion in the Unmatched Cohort

In the unmatched cohort (Table 1), perioperative blood transfusion was associated with increasing age (*p* = 0.038), lower BMI (*p* = 0.028), higher ASA grade (*p* = 0.001), mGPS of 2 (*p* < 0.001), open surgery (*p* < 0.001), and non-administration of dexamethasone (*p* < 0.001). Patients who received a perioperative blood transfusion had a significantly lower preoperative Hb (*p* < 0.001), and a significantly higher proportion had normocytic anemia (*p* < 0.001).

**Table 1:** Clinicopathological characteristics of patients undergoing elective, open surgery for colorectal cancer receiving any perioperative blood transfusion—unmatched cohort

Characteristics	All [*n *= 544]	No transfusion [*n *= 458]	Transfusion [*n *= 86]	*p* value
*Clinicopathological*				
Age, years (< 65/65–74/≥ 75)	194/208/142	168/180/110	26/28/32	0.038
Sex (male/female)	294/248	253/203	41/45	0.196
BMI (< 20/20–25/26–30/> 30 kg/m^2^)	33/169/168/164	23/142/141/145	10/27/27/19	0.028
ASA (1/2/3/4)	131/234/149/14	117/204/119/8	14/30/30/6	0.001
Iron infusion (yes/no)	19/531	18/440	1/85	0.336
Approach (laparoscopic/open)	269/263	246/200	23/63	< 0.001
Perioperative dexamethasone (yes/no)	334/138	302/104	32/34	< 0.001
Tumor site (colon/rectum)	347/196	285/173	62/23	0.065
*Operation duration, mins [median (IQR)]*				
Colonic	180 (131–252)	181 (135–240)	173 (120–207)	0.107
Rectal	275 (210–335)	275 (209–335)	283 (228–409)	0.811
TNM stage (0/I/II/III)	13/127/213/183	10/117/174/152	3/10/39/31	0.132
Neoadjuvant treatment (yes/no)	78/458	62/391	16/67	0.180
*Hematological*				
Preoperative Hb, g/L [median (IQR)]	131 (118–144)	133 (124–146)	106 (98–121)	< 0.001
Preoperative anemia (no/microcytic/normocytic)^a^	354/42/148	333/20/105	21/22/43	< 0.001
POD 1 Hb, g/L [median (IQR)]	110 (98–123)	113 (103–125)	94 (87–101)	< 0.001
POD 2 Hb, g/L [median(IQR)]	107 (97–120)	110 (100–123)	90 (82–101)	< 0.001
POD 3 Hb, g/L [median (IQR)]	108 (98–121)	111 (101–124)	95 (90–101)	< 0.001
POD 4 Hb, g/L [median (IQR)]	110 (99–122)	114 (103–125)	96 (92–105)	< 0.001
POD 5 Hb, g/L [median (IQR)]	111 (99–125)	114 (103–127)	98 (92–107)	< 0.001
*Systemic inflammation*				
Preoperative mGPS (0/1/2)^b^	399/42/56	345/37/37	54/5/19	< 0.001
POD 1 CRP, mg/L [median (IQR)]	83 (55–116)	80 (52–112)	101 (72–138)	< 0.001
POD 2 CRP, mg/L [median (IQR)]	132 (82–197)	127 (79–191)	164 (117–225)	< 0.001
POD 3 CRP, mg/L [median (IQR)]	127 (76–192)	120 (72–188)	143 (96–221)	0.004
POD 4 CRP, mg/L [median (IQR)]	101 (64–172)	100 (63–167)	115 (72–239)	0.050
POD 5 CRP, mg/L [median (IQR)]	86 (51–158)	83 (49–149)	107 (55–232)	0.051
POD 1 albumin, g/L [median (IQR)]	28 (25–31)	28 (25–31)	24 (21–26)	< 0.001
POD 2 albumin, g/L [median (IQR)]	27 (24–31)	28 (25–31)	23(21–26)	< 0.001
POD 3 albumin, g/L [median (IQR)]	26 (24–30)	27 (24–30)	24 (20–26)	< 0.001
POD 4 albumin, g/L [median (IQR)]	26 (23–29)	27 (24–30)	23 (20–26)	< 0.001
POD 5 albumin, g/L [median (IQR)]	26 (23–29)	27 (24–30)	23 (19–25)	< 0.001
poGPS 3 (0/1/2)^c^	304/115/88	262/102/62	42/13/26	0.004
poGPS 4 (0/1/2)^c^	300/62/64	249/58/41	51/4/23	0.010
*Short*-*term outcomes*				
Any complication (yes/no)	207/332	154/301	53/31	< 0.001
Infective complication (yes/no)	130/409	91/364	39/45	< 0.001
Anastomotic leak (yes/no)	23/516	8/447	15/69	< 0.001
Clavien–Dindo grade (0–2/3–5)	484/53	429/24	55/29	< 0.001
30-day mortality (yes/no)	7/532	5/449	1/83	0.451
Length of stay, days [median (IQR)]	7 (5–12)	7 (5–10)	12 (7–19)	< 0.001
*Long*-*term outcomes*				
Adjuvant treatment (yes/no)	165/295	146/240	19/55	0.048
5-year CSS (%)	86	89	68	< 0.001
5-year OS (%)	79	82	59	< 0.001

Data are expressed as *n* unless otherwise specified

*ASA* American Society of Anesthesiologists grade, *BMI* body mass index, *Hb* Hemoglobin*, CRP* C-reactive protein, *OS* overall survival, *CSS* cancer-specific survival, *POD* postoperative day, *IQR* interquartile range, *mGPS* modified Glasgow Prognostic Score, *poGPS* postoperative Glasgow Prognostic Score, *MCV* mean corpuscular volume

^a^Males = Hb < 130 g/L, females = Hb < 120 g/L; microcytic = anemia and MCV < 80 fL; normocytic = anemia and MCV 80–100 fL

^b^0 = CRP < 10 mg/L; 1 = CRP ≥ 10 mg/L and albumin ≥ 35 g/L; 2 = CRP ≥ 10 mg/L and albumin < 35 g/L

^c^0 = CRP < 150 mg/L; 1 = CRP ≥ 150 mg/L and albumin ≥ 25 g/L; 2 = CRP ≥ 150 mg/L and albumin < 25 g/L

**Fig. 1:**
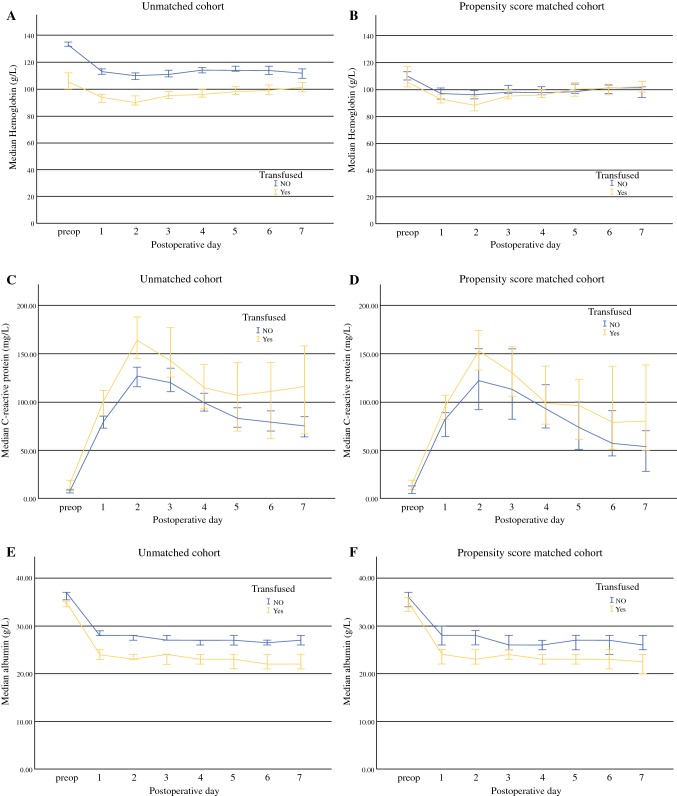
Line charts comparing patients who did (*yellow lines*) and did not (*blue lines*) receive perioperative blood transfusions with regard to postoperative median hemoglobin concentrations (g/L) in the **a** unmatched and **b** propensity score matched cohorts, postoperative median CRP concentrations (mg/L) in the **c** unmatched and **d** propensity score matched cohorts, and postoperative median albumin concentrations (g/L) in the **e** unmatched and **f** propensity score matched cohorts. Bars represent 95% confidence intervals. *preop* preoperative, *CRP* C-reactive protein

Patients who received a perioperative blood transfusion had a significantly lower Hb on PODs 1–5 (Fig. 1a), a significantly higher CRP on PODs 1–3 (Fig. 1c), and a significantly lower albumin on PODs 1–5 (Fig. 1e). A higher proportion of patients who received a perioperative blood transfusion had a poGPS of 2 on PODs 3 (32% vs. 15%; *p* = 0.004) and 4 (29% vs. 12%; *p* = 0.010).

Patients who received a perioperative blood transfusion had a significantly higher rate of postoperative complications (63% vs. 34%; *p* < 0.001), infective complications (46% vs. 20%; *p* < 0.001), anastomotic leak (18% vs. 2%; *p* < 0.001), and Clavien–Dindo grade 3–5 complications (35% vs. 5%; *p* < 0.001), as well as a longer median length of stay (12 vs. 7 days; *p* < 0.001).

Perioperative blood transfusion was significantly associated with poorer 5-year CSS (68% vs. 89%; *p* < 0.001) and poorer 5-year OS (59% vs. 82%; *p* < 0.001).

### Perioperative Blood Transfusion in the Propensity Score Matched Cohort

**Fig. 2:**
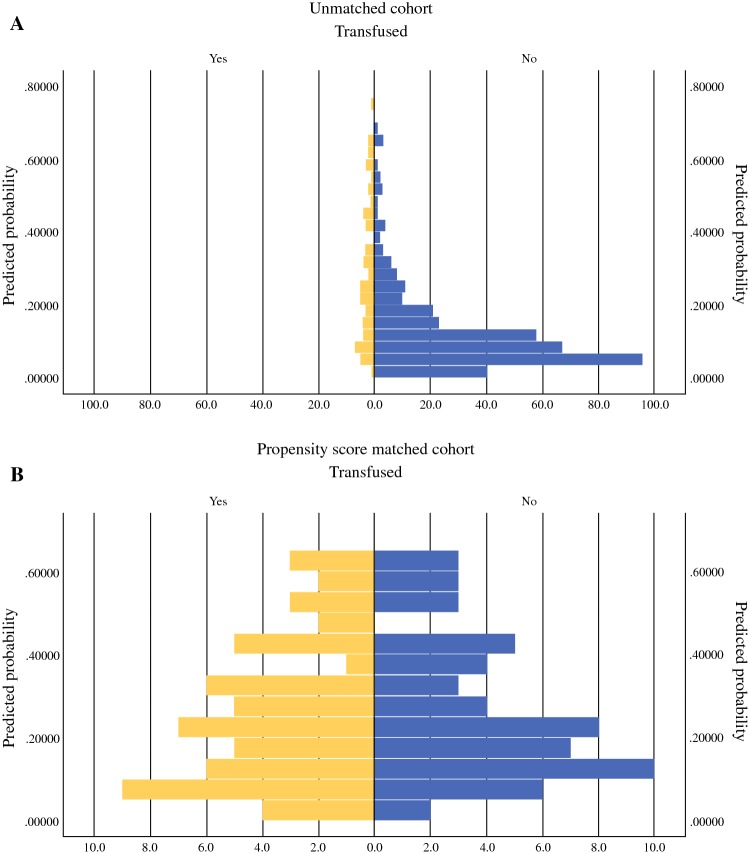
Histograms of distribution of propensity scores among patients who did and did not receive perioperative blood transfusion in the **a** unmatched cohort and **b** propensity score matched cohort

Propensity scores could not be assigned to 121 patients due to missing covariate data, leaving 423 patients with propensity scores, of whom 62 had received a perioperative blood transfusion and 361 had not (Fig. 2a). One hundred and sixteen patients were matched (58 in each group) based on their propensity scores, with a subsequent improvement in the balance of their distribution (Fig. 2b).

In the propensity score matched cohort (Table 2), patients receiving a perioperative blood transfusion had significantly lower Hb on POD 2, but at no other time point (Fig. 1b). Patients receiving a perioperative blood transfusion had a significantly higher CRP on PODs 1–4 (Fig. 1d), and significantly lower albumin on PODs 1–5 (Fig. 1f). A higher proportion of patients who received a perioperative blood transfusion had a poGPS of 2 on POD 3 (28% vs. 13%; *p* = 0.031), but not on POD 4 (*p* = 0.344).

**Table 2:** Clinicopathological characteristics of patients undergoing elective, open surgery for colorectal cancer receiving any perioperative blood transfusion—propensity score matched cohort

Characteristics	All [*n *= 116]	No transfusion [*n *= 58]	Transfusion [*n *= 58]	*p* value
*Clinicopathological*				
Age, years (< 65/65–74/≥ 75)	35/39/42	16/22/20	19/17/22	–
Sex (male/female)	62/54	32/26	30/28	–
BMI (< 20/20–25/26–30/> 30 kg/m^2^)	10/35/40/31	5/20/17/16	5/15/23/15	–
ASA (1/2/3/4)	16/49/48/3	7/26/24/1	9/23/24/2	–
Iron infusion (yes/no)	8/108	8/50	0/58	–
Approach (laparoscopic/open)	44/72	25/33	19/39	–
*Operation duration, min [median (IQR)]*				
Colonic	164 (120–204)	150 (120–193)	171 (130–210)	–
Rectal	292 (240–409)	293 (240–360)	292 (251–409)	–
Perioperative dexamethasone (yes/no)	72/44	42/16	30/28	–
Tumor site (colon/rectum)	89/27	48/10	41/17	–
TNM stage (0/I/II/III)	4/15/52/45	1/8/26/23	3/7/26/22	–
Neoadjuvant treatment (yes/no)	19/95	9/48	10/47	–
*Hematological*				
Preoperative Hb, g/L [median (IQR)]	109 (101–118)	110 (105–117)	106 (98–128)	–
Preoperative anemia (no/microcytic/normocytic)^a^	20/28/64/4	2/15/38/3	18/13/26/1	–
POD 1 Hb, g/L [median (IQR)]	95 (88–103)	97 (91–104)	93 (86–100)	0.063
POD 2 Hb, g/L [median (IQR)]	93 (84–101)	96 (90–101)	89 (82–101)	0.033
POD 3 Hb, g/L [median (IQR)]	96 (90–104)	98 (89–105)	95 (90–103)	0.443
POD 4 Hb, g/L [median (IQR)]	97 (93–106)	98 (94–107)	96 (92–106)	0.597
POD 5 Hb, g/L [median (IQR)	99 (93–107)	99 (95–108)	100 (92–107)	0.820
*Systemic inflammation*				
Preoperative mGPS (0/1/2)^b^	8/82/26	43/4/11	39/4/15	–
POD 1 CRP, mg/L [median(IQR)]	87 (64–117)	83 (55–112)	96 (71–126)	0.024
POD 2 CRP, mg/L [median(IQR)]	143 (86–198)	122 (78–183)	153 (117–201)	0.011
POD 3 CRP, mg/L [median (IQR)]	126 (81–177)	113 (66–173)	130 (93–196)	0.046
POD 4 CRP, mg/L [median (IQR)]	97 (59–174)	93 (50–157)	99 (63–239)	0.029
POD 5 CRP, mg/L [median (IQR)]	81 (41–144)	74 (42–118)	96 (40–208)	0.264
POD 1 albumin, g/L [median (IQR)]	26 (23–29)	28 (25–31)	24 (21–26)	< 0.001
POD 2 albumin, g/L [median (IQR)]	25 (23–29)	28 (24–31)	23 (21–26)	< 0.001
POD 3 albumin, g/L [median(IQR)]	25 (22–27)	26 (24–30)	24 (20–26)	< 0.001
POD 4 albumin, g/L [median (IQR)]	24 (22–27)	26 (23–29)	23 (21–26)	0.025
POD 5 albumin, g/L [median (IQR)]	24 (22–28)	27 (24–29)	23 (19–25)	0.003
poGPS 3 (0/1/2)^c^	66/21/22	35/13/7	31/8/15	0.031
poGPS 4 (0/1//2)^c^	69/8/20	32/6/7	37/2/13	0.344
*Short*-*term outcomes*				
Any complication (yes/no)	53/63	19/39	34/24	0.017
Infective complication (yes/no)	33/83	11/47	22/36	0.061
Anastomotic leak (yes/no)	10/106	1/57	9/49	0.021
Clavien–Dindo grade (0–2/3–5)	23/93	55/3	38/20	< 0.001
30-day mortality (yes/no)	1/115	0/58	1/57	–
Length of stay, days [median (IQR)]	8 (6–13)	7 (5–12)	11 (6–16)	0.011
*Long*-*term outcomes*				
Adjuvant treatment (yes/no)	32/63	19/28	13/35	0.732
5-year CSS (%)	78	90	70	0.199
5-year OS (%)	72	85	63	0.094

Data are expressed as *n* unless otherwise specified

*ASA* American Society of Anesthesiologists grade, *BMI* body mass index, *Hb* hemoglobin*, CRP* C-reactive protein, *OS* overall survival, *CSS* cancer-specific survival, *POD* postoperative day, *IQR* interquartile range, *mGPS* modified Glasgow Prognostic Score, *poGPS* postoperative Glasgow Prognostic Score, *MCV* mean corpuscular volume

^a^Males = Hb < 130 g/L, females = < 120 g/L; microcytic = anemia and MCV < 80 fL; normocytic = anemia and MCV 80–100 fL

^b^0 = CRP < 10 mg/L; 1 = CRP ≥ 10 mg/L and albumin ≥ 35 g/L; 2 = CRP ≥ 10 mg/L and albumin < 35 g/L)

^c^0 = CRP < 150 mg/L; 1 = CRP ≥ 150 mg/L and albumin ≥ 25 g/L; 2 = CRP ≥ 150 mg/L and albumin < 25 g/L

Patients who received a perioperative blood transfusion had a significantly higher rate of postoperative complications (59% vs. 33%; *p* = 0.017), anastomotic leak (15% vs. 2%; *p* = 0.021), and Clavien–Dindo grade 3–5 complications (34% vs. 5%; *p* < 0.001), as well as a longer median length of stay (11 vs. 7 days; *p* = 0.011).

There was no significant difference in 5-year CSS (%) or 5-year OS (%) between the propensity score matched groups.

### Sensitivity Analyses

A similarly statistically significant association was found at linear regression with CRP as the dependent variable and blood transfusion as the explanatory variable, in the unmatched cohort, when patients with major complications (Clavien–Dindo grades 3–5) were excluded from the analysis, when using propensity score regression, and in the propensity score matched cohort (Table 3). The same was found using binary logistic regression with postoperative complication as the outcome variable, and Cox regression with OS as the outcome variable.

**Table 3:** Sensitivity analysis: linear regression coefficients of postoperative day 3 C-reactive protein concentrations, odds ratios of postoperative complications, and hazard ratios of all-cause mortality with respect to perioperative blood transfusion across the propensity score methods

Model	*n*	POD 3 CRP [B (95% CI)]	Complication [OR (95% CI)]	Overall survival [HR (95% CI)]
Unadjusted	544	34 (15–54)	3.28 (2.03–5.29)	3.18 (2.08–4.84)
Excluding CD grade 3–5 complications	484	28 (6–51)	–	2.76 (1.65–4.66)
Propensity score regression	423	31 (8–54)	2.07 (1.14–3.77)	1.88 (1.04–3.41)
Propensity score matched	116	34 (3–64)	2.91 (1.36–6.20)	2.38 (0.99–5.73)

*B* linear regression coefficient*, POD* postoperative day*, CD* Clavien–Dindo*, CRP* C-reactive protein*, OR* odds ratio, *HR* hazard ratio, *CI* confidence interval

### Factors Associated with Poorer Outcomes Among Patients Receiving Perioperative Blood Transfusion

When those patients who received a perioperative blood transfusion (*n* = 86) were examined (Table 4), at univariate binary logistic regression, surgery for rectal cancer (*p* = 0.050), and increasing day 3 poGPS (*p* = 0.007) were associated with postoperative complications. At multivariate analysis, only day 3 poGPS remained significantly associated with postoperative complications (OR 2.47, 95% CI 1.93–3.15; *p* < 0.001).

**Table 4:** Factors associated with postoperative complications and overall survival in patients who received a perioperative blood transfusion

Postoperative complications variable	Univariate OR (95% CI)	*p* value	Multivariate OR (95% CI)	*p* value
Age	0.85 (0.50–1.46)	0.557	–	–
Male sex	1.29 (0.53–3.09)	0.575	–	–
BMI	0.85 (0.53–1.35)	0.483	–	–
ASA	1.02 (0.61–1.72)	0.928	–	–
Laparoscopic	0.70 (0.26–1.84)	0.468	–	–
Dexamethasone	0.89 (0.34–2.32)	0.806	–	–
Rectal	3.02 (1.00–9.31)	0.050	–	0.309
T stage	0.90 (0.56–1.45)	0.660	–	–
mGPS^b^	0.95 (0.57–1.61)	0.853	–	–
poGPS 3^c^	2.16 (1.24–3.76)	0.007	2.47 (1.93–3.15)	< 0.001

Overall survival variable	Univariate HR (95% CI)	*p* value	Multivariate HR (95% CI)	*p* value
Age	1.39 (0.85–2.25)	0.182	–	–
Male sex	1.41 (0.66–3.02)	0.373	–	–
BMI	0.80 (0.54–1.18)	0.251	–	–
ASA	2.19 (1.40–3.42)	0.001	1.96 (1.16–3.33)	0.012
Laparoscopic	0.44 (0.15–1.26)	0.125	–	–
Dexamethasone	0.68 (0.28–1.61)	0.378	–	–
Rectal	0.64 (0.24–1.70)	0.375	–	–
T stage	1.89 (1.07–3.36)	0.029	–	0.165
mGPS^b^	1.83 (1.18–2.84)	0.007	1.69 (1.08–2.62)	0.020
poGPS 3^c^	1.39 (0.92–2.12)	0.121	–	–
Complication	1.75 (0.77–4.01)	0.183	–	–

*ASA* American Society of Anesthesiologists grade, *BMI* body mass index, *CI* confidence interval, *HR* hazard ratio*, OS* overall survival, *mGPS* modified Glasgow Prognostic Score

^a^Males = Hb < 130 g/L, females = < 120 g/L

^b^0 = CRP < 10 mg/L; 1 = CRP ≥ 10 mg/L and albumin ≥ 35 g/L; 2 = CRP ≥ 10 mg/L and albumin < 35 g/L

^c^0 = CRP < 150 mg/L; 1 = CRP ≥ 150 mg/L and albumin ≥ 25 g/L; 2 = CRP ≥ 150 mg/L and albumin < 25 g/L

At univariate Cox regression, increasing ASA (*p* = 0.001), T stage (*p* = 0.029), and mGPS (*p* = 0.007) were significantly associated with poorer OS. At multivariate analysis, ASA (HR 1.96, 95% CI 1.16–3.33; *p* = 0.012) and mGPS (HR 1.69, 95% CI 1.08–2.62; *p* = 0.020) remained independently associated with OS.

## Discussion

The results of the present study report a significant association between perioperative blood transfusion, an exaggerated postoperative systemic inflammatory response, postoperative complications and survival following surgery for colorectal cancer. This remained the case even after propensity score matching for variables known to be associated with blood transfusion, postoperative systemic inflammatory response, and postoperative complications. Although blood transfusion is recognized to be associated with healthcare-related serious infection^19^ and short- and long-term outcomes following colorectal cancer resection, this study lends evidence to one of the proposed mechanisms—modulation of the host immune response to surgery.

There is increasing evidence that an exaggerated postoperative systemic inflammatory response is associated with postoperative complications and poorer oncologic outcomes in patients with gastrointestinal cancer.^20^–^22^ It is hypothesized that the primarily innate immune response to the trauma of surgery leads to relative suppression of adaptive responses.^10^,^23^ The reduced efficacy of this useful anticancer response, coupled with the pro-metastatic nature of the innate cellular, cytokine and neurohormonal surgical response, may increase the risk of recurrence.^24^ Furthermore, it has been observed that modulation of the postoperative systemic inflammatory response with perioperative corticosteroids is associated with fewer postoperative complications and improved survival; however, these findings are yet to be confirmed in prospective trials.^8^,^9^

Allogeneic blood transfusion in the perioperative period is widely thought to be negatively associated with short-term postoperative outcomes and survival in patients undergoing surgery for cancer, with an older Cochrane review,^4^ a recent large propensity score matched study of over 4000 patients,^25^ and a very recent comprehensive meta-analysis^3^ reporting this to be the case. However, two propensity score matched cohorts from a single group of researchers found no negative impact on survival in colon^26^ or rectal cancer,^27^ and a smaller recent observational study found no independent impact of blood transfusion on colorectal cancer recurrence when corrected for preoperative anemia.^28^ It is unclear why more recent studies tend to report no impact on survival. It may be that modern blood donation, processing, and transfusion guidelines lead to a lower negative risk profile, or it may relate to the analyses used by the reporting groups. With this in mind, the present study included preoperative anemia as a matching variable along with a wider variety of other perioperative variables, and the results are in favor of a negative impact on both short- and long-term outcomes. Given the association between perioperative blood transfusion and postoperative systemic inflammatory response, it is possible that the contribution of blood transfusion to postoperative immunologic dissonance is the mechanism by which such outcomes are affected.^29^ Indeed, this would be in keeping with older evidence reporting even poorer outcomes in patients who both received a perioperative blood transfusion and experienced a postoperative infective complication.^30^,^31^

In the propensity score matched cohort, perioperative blood transfusion was not associated with sustained significant differences in postoperative Hb. This might suggest that despite the transfusion of PRCs achieving the aim of restoring the oxygen-carrying capacity of the blood, the impact on the host immune system still leads to poorer outcome.

Alternatively, it is possible that more advanced and bulky tumors lead to longer and more difficult surgery, generating a greater inflammatory response, while also being associated with an increased transfusion requirement and a greater likelihood of morbidity and mortality. Although the propensity score matching process in the present study aimed to correct for most of these confounders, its retrospective nature prevents us from making any causal inference between transfusion and postoperative inflammatory response.

The present study has a number of limitations. Its retrospective nature leads to missing data, and the use of propensity score matching, although designed to reduce confounding, can introduce bias in parameters that have not been recorded or are unknown. Furthermore, its use leads to loss of statistical power. The lack of a formal blood transfusion protocol during the study period means that some patients with preoperative anemia did not receive a perioperative blood transfusion, whereas some patients who were not anemic did, presumably due to intraoperative blood loss or other factors. Compounding this, the small numbers of transfused patients prevented meaningful subgroup analysis of transfusion timing. Estimated blood loss was recorded very poorly, preventing this important variable from being included in the analysis. Despite this, the similar effect sizes found at sensitivity analysis suggest internal validity to the findings.

## Conclusions

The present study reports a significant association between perioperative blood transfusion, the postoperative systemic inflammatory response, complications and survival following surgery for colorectal cancer. Furthermore, in those who received a blood transfusion, an exaggerated perioperative innate systemic inflammatory response appeared to compound the negative effects on short- and long-term outcomes. Although the described relationship between blood transfusion and poorer outcomes is not new, the present study suggests a possible mechanism, i.e. modulation of the postoperative systemic inflammatory response.
